# HIV-1 subtype C superinfected individuals mount low autologous neutralizing antibody responses prior to intrasubtype superinfection

**DOI:** 10.1186/1742-4690-9-76

**Published:** 2012-09-20

**Authors:** Debby Basu, Colleen S Kraft, Megan K Murphy, Patricia J Campbell, Tianwei Yu, Peter T Hraber, Carmela Irene, Abraham Pinter, Elwyn Chomba, Joseph Mulenga, William Kilembe, Susan A Allen, Cynthia A Derdeyn, Eric Hunter

**Affiliations:** 1 Immunology and Molecular Pathogenesis Graduate Program, Emory University, Atlanta, GA, USA; 2 Department of Pathology and Laboratory Medicine, Emory University, Atlanta, GA, USA; 3 Emory Vaccine Center at Yerkes National Primate Research Center, Emory University, Atlanta, Georgia, USA; 4 Emory University, Atlanta, Georgia, USA; 5 Department of Global Health, Rollins School of Public Health, Emory University, Atlanta, Georgia, USA; 6 Los Alamos National Laboratory, Los Alamos, New Mexico, USA; 7 Public Health Research Institute Center, New Jersey Medical School, Newark, UMDNJ, New Jersey, USA; 8 Zambia Emory HIV Research Project, Lusaka, Zambia; 9 Projet San Francisco, Kigali, Rwanda; 10 Department of Pathology, Emory Vaccine Center at Yerkes National Primate Research Center, Emory University, 954 Gatewood Road, Atlanta, Georgia, 30329, USA

**Keywords:** HIV-1 superinfection, Subtype C, Neutralizing antibodies, HIV-1 transmission, HIV-1 dual infection

## Abstract

**Background:**

The potential role of antibodies in protection against intra-subtype HIV-1 superinfection remains to be understood. We compared the early neutralizing antibody (NAb) responses in three individuals, who were superinfected within one year of primary infection, to ten matched non-superinfected controls from a Zambian cohort of subtype C transmission cases. Sequence analysis of single genome amplified full-length *envs* from a previous study showed limited diversification in the individuals who became superinfected with the same HIV-1 subtype within year one post-seroconversion. We hypothesized that this reflected a blunted NAb response, which may have made these individuals more susceptible to superinfection.

**Results:**

Neutralization assays showed that autologous plasma NAb responses to the earliest, and in some cases transmitted/founder, virus were delayed and had low to undetectable titers in all three superinfected individuals prior to superinfection. In contrast, NAbs with a median IC50 titer of 1896 were detected as early as three months post-seroconversion in non-superinfected controls. Early plasma NAbs in all subjects showed limited but variable levels of heterologous neutralization breadth. Superinfected individuals also exhibited a trend toward lower levels of gp120- and V1V2-specific IgG binding antibodies but higher gp120-specific plasma IgA binding antibodies.

**Conclusions:**

These data suggest that the lack of development of IgG antibodies, as reflected in autologous NAbs as well as gp120 and V1V2 binding antibodies to the primary infection virus, combined with potentially competing, non-protective IgA antibodies, may increase susceptibility to superinfection in the context of settings where a single HIV-1 subtype predominates.

## Background

To develop a cross-protective HIV-1 vaccine that provides immunological breadth against multiple strains, a comprehensive understanding of the immunologic and virologic interactions that occur during HIV-1 superinfection in clinically relevant populations is critical. HIV-1 superinfection refers to re-infection with a heterologous HIV-1 variant in an HIV-infected individual, who has had the opportunity to mount an immune response to the primary infection [1]. Elucidating immunological factors that may prevent superinfection (despite exposure to virus) will inform our understanding of possible correlates of protection from *de novo* infection, as well as what factors may contribute towards a successful vaccine-induced immune response.

Non-human primate studies have shown that neutralizing antibodies (NAbs) and passive transfer of broadly cross-reactive monoclonal antibodies can confer protection against simian-human immunodeficiency virus (SHIV) infection [2-7]. Results of the RV144 vaccine trial have also supported that specific humoral responses, including higher levels of V1V2-binding IgG antibodies, may have contributed to protection from primary HIV-1 infection in uninfected vaccinees, and that higher anti-Env plasma IgA levels may have contributed towards risk of primary HIV-1 infection in vaccinees [8,9]. Another approach to address the potential contribution of antibodies to protection from primary HIV-1 infection is to evaluate whether they decrease susceptibility to superinfection. Specifically, antibody responses in individuals who become superinfected versus those who are similarly exposed to exogenous virus but remain singly-infected can be evaluated for differences that may confer protection. NAb responses in the context of superinfection have been studied in subtype A [10,11] and B [12,13] HIV-1 infection, in addition to settings where multiple clades and recombinant species are common [10,14]. However, to date, there is no clear resolution of whether NAbs could play a role in modulating susceptibility to superinfection or whether trends observed in such studies were context-dependent.

Studies of a commercial sex worker (CSW) cohort in Mombasa, Kenya have shown HIV-1 intra- and inter-clade superinfections to occur during both early and chronic infection [10,11,15], with no significant difference in heterologous neutralization breadth or potency against a wide panel of cross-clade pseudoviruses in superinfected individuals versus non-superinfected matched controls prior to superinfection [10]. In contrast, intrasubtype B superinfections in an MSM cohort in San Diego have been shown to occur primarily during the first year of infection, with lower baseline NAb breadth to two lab-adapted strains and autologous viruses isolated from pre-superinfection plasma [13]. Other subtype B studies have also shown, through mathematical modeling, a 21-fold reduction in the rate of superinfection after the first year of infection [16], consistent with some change in susceptibility. However, despite the fact that most new seroconversions in adults occur in heterosexual discordant couples [17] in subtype C endemic areas, the dynamics of early humoral responses in the context of superinfection in this cohort type have not been thoroughly examined.

We previously reported, from an HIV-1 discordant couple cohort in Lusaka, Zambia [17,18], a longitudinal study of 22 newly infected individuals, where three superinfection cases were identified (13.6%). HIV-1 superinfection was initially identified using a combination of screening methods with final confirmation by sequencing of single-genome amplified *env* genes [19]. In each case, superinfection was by a virus from a non-spousal partner during the first year of infection. In all cases, the superinfecting variant predominated and extensive recombination between superinfecting and initial variants occurred after the superinfection event. The finding that superinfections were commonly seen during early infection from outside partners implicated potential roles for sexual risk behavior [19] and early immune responses in modulating superinfection outcomes in this cohort. We have therefore investigated early antibody responses in these three intrasubtype C superinfected individuals and 10 of the 19 non-superinfected individuals from the same Zambian cohort of heterosexual couples.

These studies show that autologous plasma NAb titers to the early/founder viruses were low to undetectable in all three superinfected individuals prior to superinfection, whereas the majority of non-superinfected controls mounted early and strong autologous responses to the early/founder Env as early as three months post-seroconversion. Similarly, gp120 and V1V2-specific IgG antibody titers were higher in matched controls while gp120-specific plasma IgA titers were higher in two of three superinfected individuals, suggesting that reduced IgG and increased IgA humoral immune responses may influence the risk of superinfection in this subtype C cohort.

## Results

### Limited Envelope (Env) diversification in the initially infecting virus prior to superinfection

In a previous study of superinfection within a subset of 22 newly infected individuals from the Zambia-Emory HIV Research Project (ZEHRP) discordant couple cohort [19], we identified three individuals who were superinfected from non-spousal partners within the first year of infection (detected 3–10 months post-seroconversion) with subtype C superinfecting variants.

**Figure 1:**
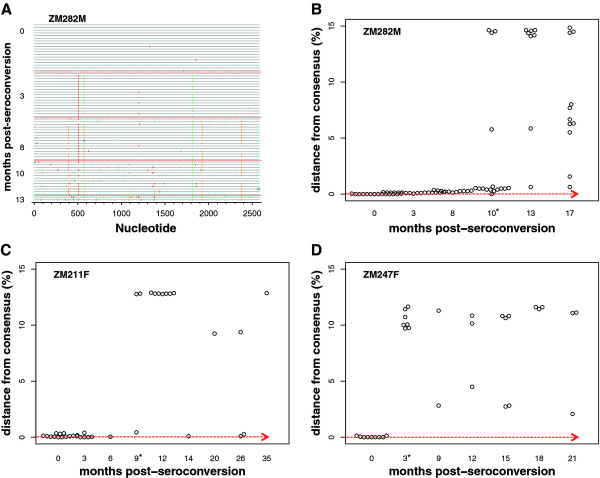
**Homogeneity of early/founder*env*sequences prior to superinfection in three intrasubtype C superinfected individuals.** Single genome amplified full-length *env* sequences were evaluated through Highlighter plots for visualization of viral evolution of early/founder variants. Nucleotide changes from the early/founder consensus *env* sequence can be visualized by the colored hatch-marks (red = T, green = A, orange = G, light blue = C) as shown with ZM282M **(A)**, in which superinfection was detected at 10 months post-seroconversion (superinfecting sequences not shown). The time point from which amplicons were isolated is shown as months after seroconversion (0-month). Raw pairwise distance from the early/founder consensus sequence to each longitudinal *env* amplicon sequence (vertical axis) was plotted for the three superinfected cases **(B-D)**. Asterisks on the x-axis indicate time at which superinfection was detected and superinfecting sequences are included, clustering at 9-15% pairwise distance from initial consensus **(B-D).**

As part of the previous study, we performed single genome amplification and sequencing of the *env* gene of the initially infecting virus at time points prior to superinfection. A phylogenetic evaluation of these longitudinal full-length *env* sequences showed remarkable homogeneity prior to superinfection for the two individuals in which superinfection was detected 9 and 10 months after primary infection. An example of this phenomenon is diagrammed via Highlighter plot for ZM282M in Figure 1A where few mutations were fixed over the first 10 months of infection. This is particularly clear when the pairwise distance of each amplicon sequence from that of the initial consensus sequence is plotted over time (Figure 1B-D). We observed a mean pairwise distance of only 0.1% prior to superinfection in both ZM282M and ZM211F, and less than 0.3% mean pairwise distance among all initially infecting variants of the three superinfected cases with respect to each individual’s initial consensus sequence. This limited diversity contrasts with previous reports of approximately 1%/year during early infection [20]. Thus, from these panels, it is evident when superinfection was detectable (x-axis asterisk, Figure 1B-D) and that there was limited *env* sequence evolution prior to this event. 

### Neutralization of early/founder viruses during early infection

**Figure 2:**
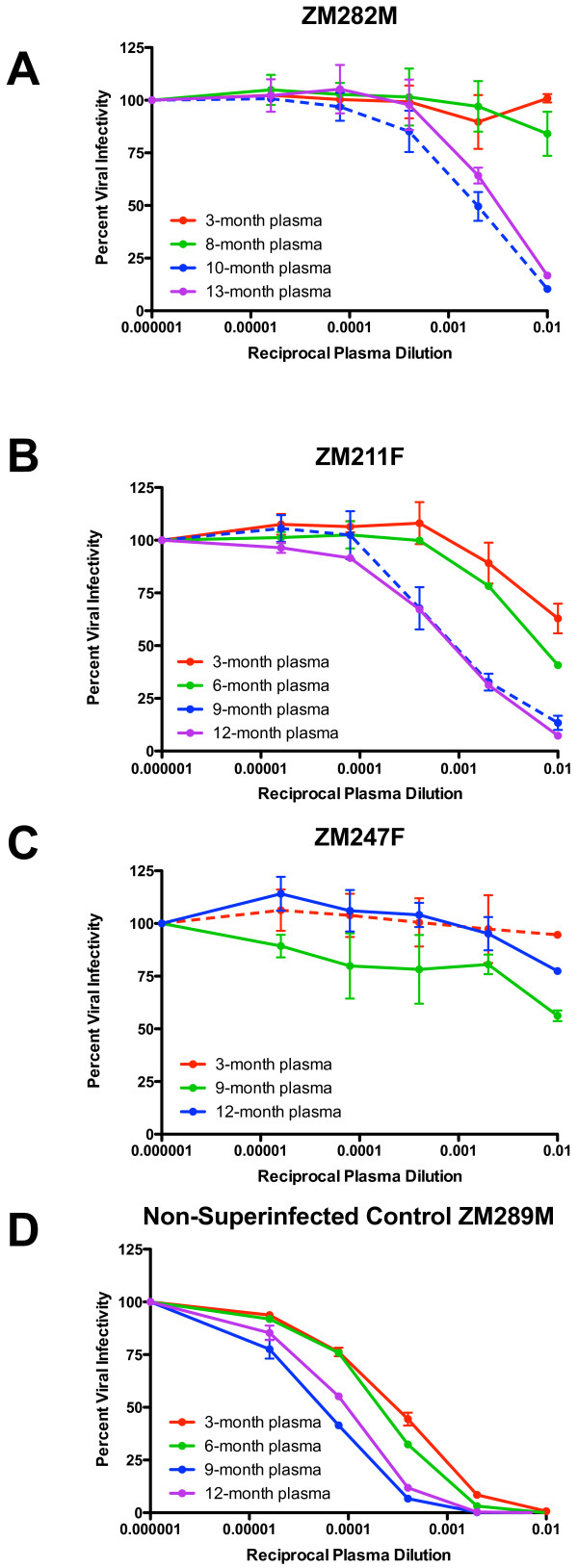
**Autologous neutralizing antibody responses to early/founder Env in superinfected individuals during early infection.** Early/founder viruses were tested for neutralization by autologous plasma from the first year of infection in superinfected **(A-C)** and non-superinfected controls (representative control shown in panel **D**). Dashed lines correspond to plasma from the time point at which superinfection was detected. Percent viral infectivity is depicted on the vertical axis, and reciprocal plasma dilution is depicted along the horizontal axis, in logarithmic fashion. Each curve represents a single plasma-virus combination, performed in duplicate wells. Error bars represent standard error of the mean between two independent experiments.

**Figure 3:**
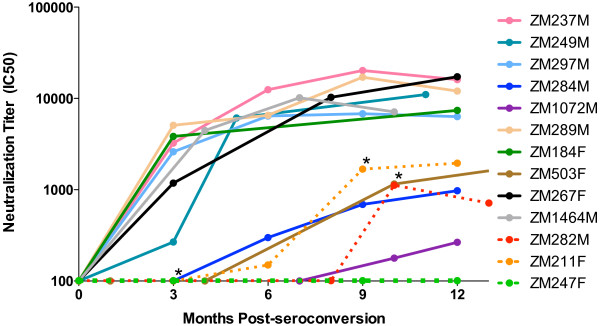
**Development of autologous neutralizing antibodies to early/founder virus Env is slow or absent prior to superinfection.** Plasma neutralizing antibody IC50 titers (representing plasma dilution necessary to achieve 50% viral infectivity) to early/founder virus Env were determined over the course of the first year of infection for three superinfected (dashed lines) and ten non-superinfected matched controls (solid lines). Values represent mean IC50 values from two independent experiments. Asterisks mark time at which superinfection was detected in the superinfected cases.

To evaluate early NAb responses in the three superinfected individuals, we matched the superinfected individuals to 10 of the 19 newly-infected non-superinfected unlinked partners. Matching parameters included subtype of infection, viral load at time of seroconversion, estimated time to infection, sample dates available, seroconversion in the same 5-year time span, and when possible, individuals self-reporting outside partnerships were included (Table 1). To test the hypothesis that lower titers of autologous NAb to the initial infection (early/founder Env) exist in the superinfected individuals, we utilized single genome amplification to obtain an average of 10 *env* amplicons (range 8–12) per individual (Additional file 1: Figure S1). After cloning of amplicons representing the consensus early/founder sequence (Table 1), we generated pseudoviruses carrying these Env glycoproteins for the three superinfected and the 10 singly-infected individuals. Using the standard JC53BL-13 (TZM-bl) neutralization assay [21-25], we tested autologous neutralization of the early/founder Envs by longitudinal plasma samples from the first year of infection. Viral infectivity curves plotting autologous neutralization of these early/founder variants were generated for each plasma time-point for each individual. Examples of these for the superinfected individuals and one non-superinfected control are shown in Figure 2A-D. Using these curves, plasma neutralizing antibody IC50 titers, which represent the plasma dilutions at which 50% of viral infectivities is achieved, were determined [22] over the course of the first year of infection for superinfected (Figure 3, dashed lines) and non-superinfected (non-SI) matched controls (Figure 3, solid lines). 

**Table 1:** Seroconverters from ZEHRP cohort evaluated for longitudinal autologous neutralization of initial variants

**Subject ID**	**Linkage**	**Subtype**	**Last Seronegative Date**	**First Sample Date**	**Estimated days from infection**^**a**^	**Viral load**^**b**^	**Setpoint Viral load**^**c**^	**Estimated timing of SI**^**d**^	**Viral load at SI**^**e**^	**Sex with Condom**^**f**^	**Sex without Condom**^**f**^	**Outside Partner**^**g**^	**Initial Virus Env**^**h**^
**ZM282M**	**Unlinked**	**C**	**8-Dec-04**	**3-Mar-05**	**43**	**>750,0000**	**253,000**	**10**	**300,0000**	**78**	**2**	**Yes**	**Founder**
**ZM211F**	**Unlinked**	**C**	**15-Mar-02**	**5-Jul-02**	**60**	**2640**	**14,454**	**9**	**14,454**	**11**	**3**	**No**	**Early**
**ZM247F**	**Unlinked**	**C**	**29-Jul-03**	**1-Nov-03**	**26**	**>750,000**	**67,472**	**3**	**43,428**	**112**	**26**	**No**	**Founder**
ZM1072M	Unlinked	C	19-May-06	16-Aug-06	45	36,200	15,400	N/A	N/A	144	0	Yes	Founder
ZM297M	Unlinked	C	4-Mar-05	9-Jun-05	49	8,687	45,500	N/A	N/A	108	3	No	Early
ZM1464M	Unlinked	C	9-Dec-06	10-Mar-07	46	44,500	15,600	N/A	N/A	68	0	No	Early
ZM503F	Unlinked	C	2-Nov-06	9-Feb-07	50	25,800	17,100	N/A	N/A	65	0	No	Founder
**ZM249M**	**Unlinked**	**C**	**6-May-03**	**12-Aug-03**	**29**	**>750,000**	**82,233**	**N/A**	**N/A**	**145**	**25**	**Yes**	**Founder**
ZM267F	Unlinked	C	23-Mar-04	29-Jun-04	29	>750,000	50,061	N/A	N/A	188	8	No	Founder
ZM284M	Unlinked	C	14-Jan-05	9-Apr-05	22	>750,000	354,880	N/A	N/A	97	1	No	Early
ZM289M	Unlinked	C	30-Apr-05	19-May-05	41	399,737	4,512	N/A	N/A	75	8	No	Founder
ZM237M	Unlinked	C	29-Apr-03	29-Jul-03	46	44,870	59,250	N/A	N/A	41	4	No	Early
**ZM184F**	**Unlinked**	**C**	**11-Apr-03**	**10-Jul-03**	**45**	**71,290**	**72,718**	**N/A**	**N/A**	**39**	**9**	**Yes**	**Early**

Bolded subjects: superinfected individuals with evidence of superinfection from outsider partnerships or non-superinfected controls who self-reported outside partnerships.

^a^ Days from time of infection to first sample date was calculated, as follows: If only first Ab+ dates were available, time of infection was determined by the midpoint between last seronegative and first Ab+ date. If Ag+Ab- dates were available, time of infection was estimated by subtracting 22 days from Ag+Ab- dates. (Haaland 2009).

^b^ Viral load: RNA copies/ml in plasma at first sample date.

^c^ Viral load: RNA copies/ml in plasma at 7-12 months post-seroconversion.

^d^ Months post-seroconversion in which superinfection was detected, SI = Superinfection, N/A = Not Applicable.

^e^ Viral load: RNA copies/ml in plasma at time of superinfection detection.

^f^ Sexual data is cumulative events for 12 months (Kraft 2012).

^g^ Other partners self-reported for 12 months (Kraft 2012).

^h^ Denotes if initial virus envelope (Env) tested represents a transmitted/founder Env or an early founder-like Env isolated from first sample date.

**Figure 4:**
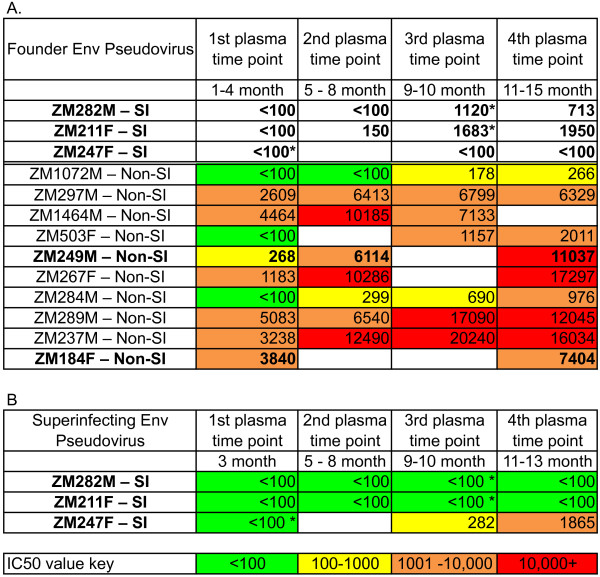
**Summary of neutralization titers to initial and superinfecting variants.** Plasma neutralizing antibody IC50 titers to the transmitted founder virus Env were determined over the course of the first year of infection for three superinfected (ZM282M, ZM211F and ZM247F, bolded) and ten non-superinfected (non-SI) case-matched controls **(A)**. Non-superinfected controls that had self-reported outside partnerships are also bolded. Similarly, IC50s to superinfecting variants were determined over the course of the first year of infection for all three superinfected cases **(B)**. Values represent mean IC50 values from two independent experiments. Asterisks mark time in which superinfection was detected in the superinfected cases.

Intrasubtype C superinfected individuals showed delayed kinetics and low-titer autologous NAb responses to the early/founder Env prior to detection of superinfection as compared to a majority of the non-superinfected controls, which had a median IC50 of 1896 as early as three-months post-seroconversion (Figure 3). Neutralization IC50 titers in the superinfected group were significantly lower at the pre-superinfection window of 5–8 months post-seroconversion compared to non-superinfected controls (p = 0.039). Although variable, neutralization kinetics and potency in the controls are similar to what has previously been shown [22,26], and are, therefore, an appropriate representation of typical early neutralization trends of subtype C infected seroconverters. A summary table (Figure 4A) of these autologous NAb IC50 titers highlights the early and strong responses seen in most non-superinfected controls, and the slower, low responses in superinfected individuals prior to superinfection. Non-superinfected controls that self-reported outside partnerships are bolded (Table 1 and Figure 4A). 

Interestingly, IC50 titers in ZM211F and ZM282M, in which superinfection was detected at 9 and 10-months post-seroconversion, were very low (150 and <100, respectively) until the time point at which superinfection was detected (dashed line, Figure 2; asterisk, Figure 3), suggesting that infection with a distinct secondary variant may have elicited an immunological boost. In ZM247F, in which we detected superinfection at 3-months and an early predominance of the superinfecting variant [19], we could not detect titers of neutralizing antibodies greater than 100 to a founder variant even at 12 months (Figure 3); it was not until 15-months post-seroconversion, when evidence for re-emergence of the founder virus was observed [19], that neutralizing antibodies to the founder became measurable (IC50 of 1092; Additional file 1: Figure S2A).

### Cross-neutralization of superinfecting viruses during early infection

To investigate further possible reasons for susceptibility to superinfection, we determined whether pre-superinfection plasma was capable of cross-neutralizing pseudoviruses carrying Env glycoproteins isolated from the time at which superinfection was first detected. No evidence for cross-neutralization of the superinfecting variants by autologous pre-superinfection plasma existed for any of the three cases (Figure 4B). However, these superinfecting variants were neutralized by pooled subtype C plasma with IC50s of 210–572, suggesting they are not inherently neutralization resistant (Additional file 1: Figure S3).

In the case of early superinfection in ZM247F, although neutralization of the founder Env was not observed until after the first year of infection (Additional file 1: Figure S2A), we did observe preferential neutralization of the superinfecting variant with titers increasing from 6–12 months after superinfection was detected (Additional file 1: Figure S2B). This likely reflects the predominance of the superinfecting virus from 3–12 months post-seroconversion [19].

### Heterologous neutralization breadth potential prior to superinfection

To evaluate whether superinfected individuals also lacked cross-neutralizing antibody breadth, we determined the ability of pre-superinfection plasma (as compared to contemporaneous plasma from controls) from early infection to neutralize a subtype C reference panel of 12 pseudoviruses. This panel included envelopes with tier 1 (“easiest” to neutralize) and tier 2 (more difficult to neutralize) sensitivities [27,28].

**Figure 5:**
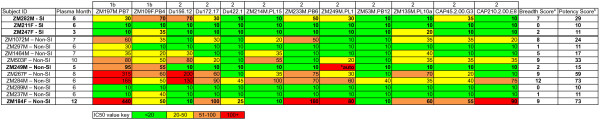
**Cross-neutralizing Breadth and Potency against HIV-1 Subtype C Env Reference Panel.** Bolded individuals represent superinfected individuals with evidence of superinfection from outsider partnerships (ZM282M, ZM211F and ZM247F) or non-superinfected controls who self-reported outside partnerships. Pre-superinfection plasma for superinfected individuals or similar plasma time points for non-superinfected controls was tested against a panel of twelve Subtype C envelope pseudoviruses. This panel included Envs of both Tier 1b and Tier 2 sensitivities. Starting plasma dilution was reduced to 1:20 to increase assay sensitivity. Plasma-env combinations, which did not reach an IC50 value at the lowest dilution tested (1:20), were assigned a value of 10. Breadth score was calculated by adding the total number of envelopes neutralized at an IC50 greater than or equal to 20. Potency score was calculated by dividing individual plasma-env IC50 by median IC50 per envelope against all plasma and then adding the sum of these scores (rounded to the nearest integer) for each plasma. “Auto” indicates that a plasma sample was tested against an autologous envelope in the panel, autologous IC50 values were not counted in breadth and potency scores.

For these studies, the lowest plasma dilution was decreased to 1:20 to increase sensitivity of the assay. Pre-superinfection 6-month plasma from ZM211F was not able to neutralize to 50% any of the subtype C pseudoviruses tested (Additional file 1: Figure S4B, Figure 5). ZM247F 3-month plasma was capable of cross-neutralizing two pseudoviruses at very low IC50s (20 and 35) and ZM282M 8-month plasma cross-neutralized seven pseudoviruses at IC50s greater than 20 but less than 100 (Additional file 1: Figure S4C and 4A, Figure 5). Non-superinfected controls showed similarly limited capacity for cross-neutralization (Figure 5). Interestingly, three non-superinfected controls (ZM284M, ZM503F, ZM1072M) that had the lowest autologous titers over the first year (Figure 4A), had some of the widest cross-neutralizing capabilities (Figure 5), indicating that heterologous breadth and autologous neutralization are not always correlated, consistent with previously published results from this cohort [22]. 

### Analysis of gp120 and V1V2-loop binding antibody levels prior to superinfection

**Figure 6:**
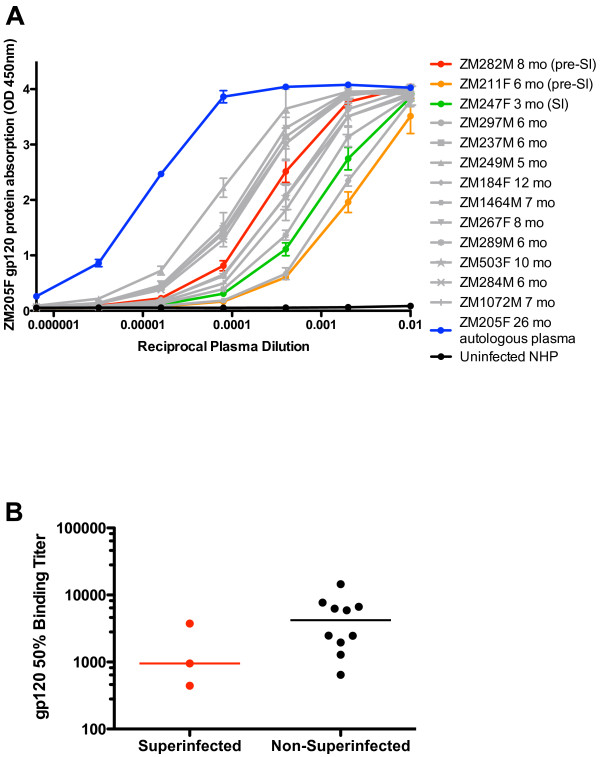
**Plasma IgG binding antibody levels to purified subtype C gp120 protein is also reduced in superinfected individuals.** Purified gp120 protein from the Zambian subtype C seroconverter ZM205F [23,29] was used with serial dilutions of plasma in a gp120 binding ELISA. Autologous plasma from ZM205F was used as a positive control for presence of gp120-specific binding antibodies (blue). Levels of gp120-specific IgG binding antibodies in plasma from time points prior to superinfection for superinfected individuals (ZM282M: red, ZM211F: orange) and similar plasma time points for non-superinfected controls (grey) was measured as shown in panel **A**. For ZM247F, in which superinfection was detected at 3-months post-seroconversion, we tested this 3-month plasma (green). Values for 50% gp120 binding in this assay were determined and compared between superinfected and non-superinfected groups **(B)** using both a mixed-linear effects model (p = 0.115) and a Mann–Whitney test to compare medians between the groups (p = 0.161).

Recent analyses of the RV144 vaccine trial in Thailand have implicated non-neutralizing antibodies in protection from acquisition of HIV-1. We therefore measured levels of gp120-specific binding IgG antibodies in pre-superinfection plasma for superinfected individuals (Figure 6A; ZM282M: red, ZM211F: orange, ZM247F: green) and similar time points for non-superinfected controls (grey). Log_10_ values for 50% of maximum gp120 binding in this assay were determined and compared between superinfected and non-superinfected groups using a mixed linear effects model. While the plasma from superinfected individuals trended to lower titers, this was not significant (p = 0.115). Median values for 50% gp120-binding between the groups was also compared by Mann–Whitney test and showed similar results (Figure 6B, p = 0.161).

**Figure 7:**
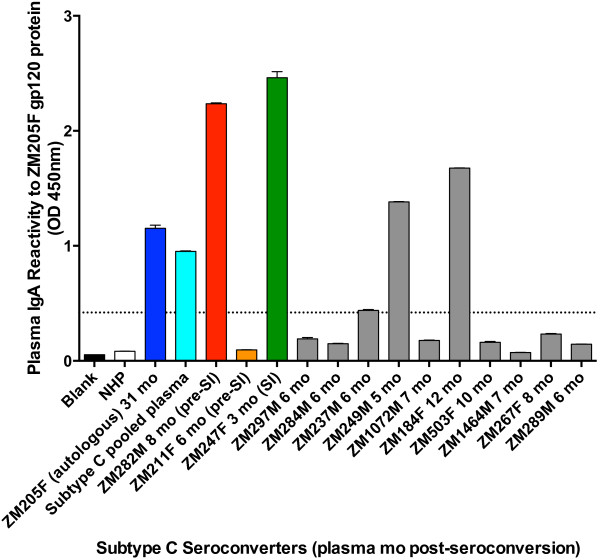
**Plasma IgA levels to purified subtype C gp120 protein are highest in two of the three superinfected individuals.** Purified gp120 protein from the Zambian subtype C seroconverter ZM205F [23,29] was used with serial dilutions of IgG-depleted plasma in a gp120 binding ELISA. Autologous plasma from ZM205F was used as a positive control for presence of gp120-specific binding antibodies (dark blue). Levels of gp120-specific IgA binding antibodies in IgG-depleted plasma from time points prior to superinfection for superinfected individuals (ZM282M: red, ZM211F: orange) and similar plasma time points for non-superinfected controls (grey) was measured at a 1:125 plasma dilution. For ZM247F, in which superinfection was detected at 3-months post-seroconversion, we tested this 3-month plasma (green). Positive absorption was recognized as absorption greater than five-times that of the normal human plasma (NHP) control and is shown as a dashed line.

We also evaluated differences in gp120-specific plasma IgA levels in pre-superinfection plasma in superinfected individuals versus similar time points for matched controls, since high plasma IgA levels were correlated with risk of HIV-1 infection in the RV144 trial [8]. Strikingly, two of the three superinfected individuals had the highest levels of plasma IgA amongst all study participants (Figure 7). Only two of the ten non-superinfected controls elicited these gp120-specific plasma IgA responses during early infection. These two matched controls were also the only non-superinfected individuals that had self-reported outside partnerships (a risk indicator of sexual exposure). When we compared the median absorption values between groups based on sexual exposure (superinfected individuals and non-superinfected individuals with self-reported outside partnerships against the non-superinfected individuals without self-reported outside partnerships) using a Mann–Whitney test we found that there was a statistically significant difference in plasma IgA levels between the groups (p = 0.005). 

**Figure 8:**
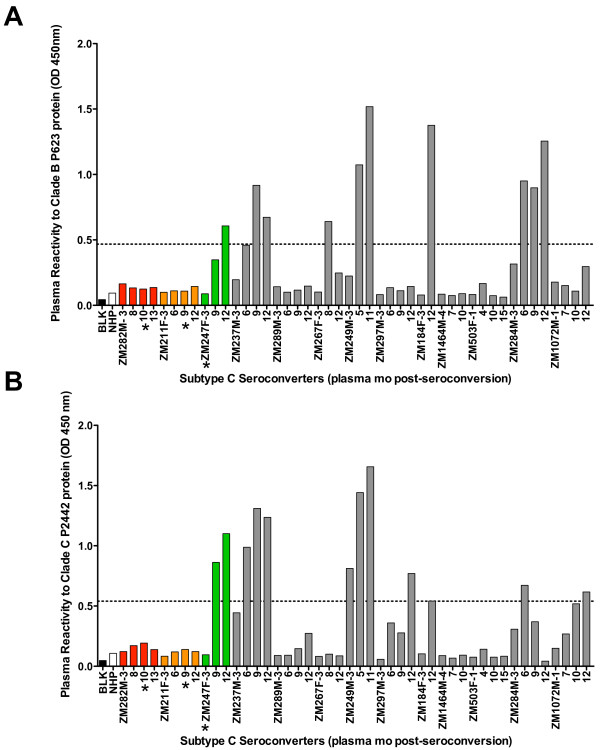
**Plasma binding antibodies to both clade B and C gp120 V1V2-loop proteins are absent in superinfected individuals prior to superinfection.** Plasma reactivity (at a single 1:500 dilution) to both P623 MuLVgp70-caseA2clBV1V2 [30]**(A)** and P2442 MuLVgp70-consensus clade C V1V2 **(B)** proteins was measured in a standard ELISA assay. Longitudinal plasma from the first year of infection in both superinfected (colored) and non-superinfected (grey) controls was tested. Asterisks denote time at which superinfection was detected. Positive absorption was recognized as absorption greater than five-times that of the normal human plasma (NHP) control and is shown as a dashed line. Figure is representative of two independent experiments.

IgG antibodies binding to the V1V2-loop of gp120, which were correlated with protection in the RV144 vaccine trial [8,9], were also quantitated using the same MuLV gp70-V1V2 construct used in that study [30] as shown in Figure 8A. Plasma reactivity to a MuLVgp70-consensus clade C V1V2 construct was also tested (Figure 8B). None of the superinfected individuals showed evidence of binding antibodies to either V1V2 construct prior to or at the time of superinfection. In contrast, three of the ten non-superinfected controls showed evidence of antibodies capable of binding both constructs within the first 6 months of infection, with seven of the ten non-superinfected plasma samples binding to at least one V1V2 protein. 

## Discussion

In this study, we have shown that three intrasubtype C superinfected individuals, in whom superinfection was detected within the first year of infection, have low to undetectable titers of autologous NAbs to their early/founder Env prior to superinfection and as late as 8-months post-seroconversion. This is in sharp contrast to ten matched non-superinfected controls similarly evaluated for neutralization of early/founder variants over the first year of infection, of which a majority mounted very potent neutralizing activities. This occurred as early as three-months post-seroconversion, when the median IC50 was 1896. Despite the small size of this study, the differences in autologous NAb titers were significantly different between the two groups (p = 0.039), and suggest that slower development of a humoral immune response increased susceptibility to intra-subtype superinfection in this cohort.

This result is consistent with a previous study of a subtype B MSM cohort, where low titers of autologous and heterologous NAbs were observed in the three superinfected individuals relative to matched non-superinfected controls [13]. However in this same study, autologous pre-superinfection Envs were tested for neutralization only cross-sectionally against contemporaneous pre-superinfection and post-superinfection plasma, and heterologous breadth assays were performed against only two lab-adapted subtype B strains. Moreover, there was no evaluation of cross-neutralization of the superinfecting virus using plasma prior to superinfection [13]. Nevertheless, there is a common observation that superinfection occurred during the first year of infection, and was associated with low autologous neutralizing antibody responses [13]. These results are consistent with the hypothesis that higher susceptibility to superinfection during early infection may be, in part, due to diminished early humoral responses.

A different conclusion was reached from a study of superinfection in HIV-1 infected commercial sex workers in Mombasa, Kenya [10]. There it was shown that while NAb breadth and potency were lower in superinfected individuals than in matched controls after approximately one year of infection, no difference in these parameters occurred immediately prior to superinfection (between 0.72-5 years post-infection) [10]. In 4/6 cases identified in that study, superinfection occurred at or after two years of the initial infection, potentially allowing for development of stronger, yet still not protective, NAb responses [10]. Thus in this multiple HIV-1 subtype sex worker cohort, NAb did not appear to provide any protection from superinfection. While the authors did not investigate autologous NAb responses to transmitted/founder Env glycoproteins in the study, responses to initial variants cloned from the time of superinfection detection and early Envs from within the first year of infection were evaluated [10].

To evaluate cross-neutralization breadth prior to superinfection, we evaluated the potential of pre-superinfection plasma to neutralize not only superinfecting variants, isolated at the time superinfection was detected, but also a subtype C reference panel of pseudoviruses. We found that pre-superinfection plasma was unable to neutralize superinfecting variants and had limited ability to cross-neutralize a panel of variants prior to superinfection, with a range of 0–7 (of 12) variants neutralized at very low IC50s (20–70) amongst all three superinfected cases. Heterologous breadth in non-superinfected control plasma samples was similarly limited, though some individuals did have greater breadth but not potency. These data are consistent with previous studies, which showed that early autologous NAbs in subtype C infection are monotypic with limited cross-neutralization potential [22,23,26,31,32]. Furthermore, it has been demonstrated that significant cross-neutralizing antibody breadth is unlikely to occur prior to chronic infection [33,34].

Heterologous neutralizing antibody breadth did not necessarily correlate with strength or effectiveness of autologous NAb responses. Although some non-superinfected individuals clearly mount strong autologous responses, they may exhibit limited neutralizing breadth by primarily targeting single or nonconserved epitopes [22,23,25,26,31,32,35,36]. In contrast, others with relatively low-titer autologous responses may in fact have wider breadth to multiple epitopes (or different epitopes), none of which confers a particularly effective neutralizing antibody response to the established infecting variant. Thus, this study suggests that, in the context of intrasubtype superinfections, either the ability to potently neutralize autologous virus or to target multiple epitopes could provide protection against superinfection. However, in the absence of both of these humoral responses, individuals may be predisposed towards superinfection.

Based on the data suggesting early deficits in NAb responses in superinfected individuals, but with little evidence for broadly neutralizing antibodies in the matched controls, we investigated whether levels of non-neutralizing antibodies also differed in the two groups prior to superinfection. We observed that superinfected individuals trended towards having lower levels of gp120-specific IgG antibodies prior to superinfection compared to controls, although this comparison did not achieve statistical significance (p = 0.115).

Similarly, we observed no reactivity to either consensus clade C or caseA2clB (clade B) V1V2-loop fusion proteins [8,30] in plasma from superinfected individuals prior to superinfection. By contrast in 3/10 non-superinfected matched controls, we observed reactivity to both proteins during the first 6 months, and in 6/10 controls reactivity was seen against the consensus C protein during the first year of infection. Higher levels of IgG V1V2-loop binding antibodies have been correlated with protection from primary HIV-1 infection in vaccinees that remained uninfected in the RV144 trial [8,9], and the data presented here are consistent with the concept that these antibodies may contribute toward protection in individuals that remained only singly-infected.

In the RV144 trial, levels of IgA antibodies capable of binding to gp120 were directly correlated with the risk of infection [8,9]. It is of interest, therefore, that two of the three superinfected individuals showed the highest anti-gp120 plasma IgA levels amongst all study participants, while only two of the ten matched controls demonstrated positive IgA binding titers. One superinfected individual, ZM211F, showed no evidence of anti-gp120 IgA reactivity. However, this is consistent with the low overall HIV-1 specific humoral responses observed, including the lowest levels of V1V2-loop and gp120-specific IgG binding antibodies prior to superinfection. We have also found a statistically significant difference in anti-gp120 plasma IgA levels with respect to sexual exposure and potential HIV-1 acquisition risk, in that individuals either with superinfection (as a result of outside partnerships) or self-reported outside partnerships (in non-superinfected individuals) had significantly higher anti-gp120 plasma IgA responses (p = 0.005), as compared to non-superinfected controls without self-reported outside partnerships. This data corroborates those drawn from the RV144 trial that high plasma IgA levels may be a surrogate of HIV-1 exposure or a potential correlate of risk in the context of primary HIV-1 infection [8] and superinfection. We have yet to evaluate the mechanism by which these differences in plasma IgA levels may affect susceptibility to infection, however it has been suggested that high levels of IgA may interfere with other potentially protective antibody-mediated effector functions such as antibody-dependent cellular cytotoxicity (ADCC) [8]. Non-neutralizing IgG antibodies could play a major role in increased mucosal barrier protection, sequestering the virus at the epithelial surface and in female genital tract mucus and contributing to Fc receptor-mediated antiviral activity [6,37,38]. Thus a diminished non-neutralizing IgG antibody response, compounded by potentially interfering IgA antibodies, could lead to reduced mucosal protection and higher susceptibility to superinfection. Future studies will elucidate whether non-neutralizing antibody-mediated antiviral activities contribute to protection from superinfection.

## Conclusions

Our previous study demonstrated three intrasubtype C superinfections during the first year of infection, with no evidence of superinfection beyond year one in 19 individuals, despite longitudinal follow-up for more than three years [19]. This observation suggested that the risk of superinfection may be highest during the first year of infection, as has been predicted by mathematical modeling in a subtype B cohort [16]. Here we evaluated the potential of the humoral immune response in natural infection to protect against superinfection. Autologous NAb responses were markedly delayed and lower in magnitude in superinfected individuals prior to superinfection detection (p = 0.039). Because of the strain-specific nature of early autologous NAb, this difference in titers may be a surrogate marker for a potential immunological deficit in protective antibodies or another factor contributing to effective humoral responses. Nevertheless, if confirmed in a larger ongoing study, these data provide support for the feasibility of inducing a protective immune response via an HIV-1 vaccine, in regions where subtype diversity is limited. It will be critical to understand the nature of vaccine-induced humoral responses and to what degree these antibodies can protect from *de novo* infection.

## Methods

### Study subjects

Heterosexual cohabiting couples in serodiscordant relationships were followed by the Zambia-Emory HIV Research Project (ZEHRP) in Lusaka, Zambia. ZEHRP provides couples’ voluntary counseling and testing as well as condom provision, general health care, and family planning counseling to participating couples [18,39]. These strategies have been shown to effectively reduce transmission rates between partners in participating countries [17]. Couples are tested for HIV-positivity, as previously published [18,39-41]. Seroconversion of the initially uninfected partner occurs approximately 7-8% per year, and the new seroconverter is subsequently followed quarterly, with annual follow-up for the chronic partner. Plasma is collected at each visit from study participants, with informed consent and under human subject protocols approved by both the University of Zambia Research Ethics Committee and the Emory University Institutional Review Board.

Epidemiological linkage was determined as previously published; unlinked transmission pairs, in which the negative partner in the serodiscordant couple became infected from an individual outside of the partnership, were identified [42]. Twenty-two unlinked couples were chosen and screened for superinfection for up to 5.5 years of follow-up [19]. Viral RNA was extracted from plasma samples at the time of seroconversion and longitudinally thereafter using the QiaAMP Viral Mini Extraction kit for phylogenetic evaluation of viral sequences. Identified superinfected individuals were matched to 10 selected non-superinfected controls from the study [19] based on 1) time from the last seronegative to the first antigen or antibody positive sample, 2) seroconversion viral load, 3) subtype of infection and 4) occurrence of seroconversions within the same five-year interval. When possible, superinfected controls were matched to non-superinfected controls that had self-reported extra-marital (outside) partnerships (ZM249M, ZM184F). Underreporting of sexual exposures is common in this Zambian cohort [43], and in this study, all initial infections were identified as being epidemiologically unlinked, implicating risk for outside partnerships despite lack of self-reported cases.

### Superinfection detection and characterization

Superinfections were identified by a combination of screening methods including phylogenetic analysis of gp41 and p24 gag population sequences, heteroduplex mobility assays using gp41 amplicons, and degenerate base counting of population sequences [19]. If individuals showed preliminary evidence of superinfection, longitudinal full-length *env* single genome amplification was performed using nested PCR [19,29,44,45] in order to confirm the presence of superinfection by phylogenetic analysis [19]. Sanger DNA sequencing was performed by the University of Alabama at Birmingham Center for AIDS Research (P30 A127767) DNA Sequencing Shared Facility using a 3730xl DNA Analyzer and BigDye Terminator v3.1 chemistry.

### Phylogenetic analysis

Sequences were assembled and evaluated using Sequencher 4.10 (Gene Codes Corporation, Ann Arbor, MI) and Geneious Pro 5.4.6 (Biomatters Ltd, Auckland, New Zealand). Sequence alignments and neighbor-joining phylogenetic trees were generated using the Tamura-Nei genetic distance model with the bootstrap resampling method. Superinfecting variant gp41 sequences consistently had greater than 11% pairwise distance from the initial founder virus [19]. Single genome sequences of full-length *envs* were amplified from plasma samples from all superinfected individuals from the time of seroconversion and longitudinally for the first year. All *envs* were processed as described above for phylogenetic analysis. Highlighter plots (LANL HIV Sequence Database) were used to evaluate longitudinal evolution of full-length *env* sequences from the determined early/founder sequence (see below). These plots were generated using aligned nucleotide sequences of the initial infection sequences only; each colored hatch-mark represents a single nucleotide change from the early/founder *env* sequence (red = T, green = A, orange = G, light blue = C). For longitudinal pairwise distance analyses, we used codon-aligned sequences to generate seroconversion consensus sequences, then computed raw pairwise distances to this consensus for each sequence sampled using R (version 2.15.0) with the package ‘ape’ (version 3.0-3) [46,47].

### Amplification and cloning of full-length *env* genes

Phusion HotStart II Hi-Fidelity DNA polymerase (Finnzymes, Thermo Scientific) was used to amplify an average of 10 single genome full-length *env* amplicons per subject from plasma by nested PCR, as described elsewhere [45]. These amplicons were obtained from the time of seroconversion (Table 1, Additional file 1: Figure S1), and a sequence alignment was generated to establish the consensus from this time point. The amplicons whose sequences matched this consensus were typically representative of the founder virus envelope glycoproteins (Table 1, Additional file 1: Figure S1). Superinfecting virus amplicons were selected by comparing the chosen early/founder virus sequence against all *env* sequences at the time of superinfection detection and selecting the superinfecting *env* variant with the greatest pairwise distance from the early/founder virus *env* sequence [19]. These *env* genes were then directionally cloned using the pcDNA 3.1 Directional TOPO/v-His expression plasmid (Invitrogen), as previously described [21,22,29]. All clones were tested for biological function, sequenced, and co-transfected with *env*-defective subtype B provirus SG3ΔEnv into 293 T cells using FuGENE HD (Roche) to generate envelope pseudoviruses carrying patient-derived *env* genes [21-24,29]. Envelope pseudoviruses were harvested 48-hours post-transfection. JC53BL-13 (TZM-bl) cells were infected at five-fold serial dilutions of virus for 48 hours for viral titering, and infectious units were determined through *β*-galactosidase staining and counting positive infected blue foci, as previously described [21-24,29].

### Neutralization assays & calculation of IC50 titers

Neutralization assays using the indicator cell line JC53BL-13 (TZM-bl) were performed, as previously described [21-25,29]. Briefly, two thousand infectious units of envelope pseudoviruses in 3.5% FBS DMEM with 40 μg/ml DEAE-Dextran were incubated with five-fold serial dilutions of heat-inactivated patient plasma. Normal human plasma (NHP) was used to maintain an overall 10% concentration of plasma [22,25,29]. The virus-plasma mixture was added to seeded JC53BL-13 cells (plated and cultured overnight at 37°C) and incubated at 37°C for 48 hours, as previously described [22-25,29]. Cells were then lysed and luciferase was read for each well; luminescence was recorded accounting for background. Percent viral infectivity and correlating neutralization IC50 values (representing plasma dilution resulting in 50% viral infectivity) were determined using a linear-regression-least squares fit method, as described elsewhere [21-24,29]. For example, if 50% viral infectivity was achieved with a 1:2000 plasma dilution, the reported IC50 would be 2000. Each plasma-Env combination was tested in duplicate in each experiment and IC50 values shown represent mean IC50 values from at least two independent experiments.

For studies of autologous plasma neutralization, plasma dilutions started at 1:100, while for heterologous breadth studies, we started at a 1:20 plasma dilution in order to increase sensitivity for low titers of cross-reactive neutralizing antibodies. For the autologous neutralization studies, we tested the first post-seroconversion plasma and all subsequent plasma samples available within approximately the first year of infection. Seroconversion plasma was not tested for neutralization, and was assumed to be at our limit of detection for these studies at an IC50 of 100.

### Heterologous neutralizing breadth and potency scores

The Subtype C HIV-1 Reference Panel of Env Clones [27,28] was obtained from NIH AIDS Reference and Reagent Program, and pre-superinfection plasma in superinfected individuals (and contemporaneous samples from non-superinfected controls) was evaluated for heterologous breadth to the 12-pseudovirus panel. After generating viral infectivity curves, neutralization IC50 values were calculated for each plasma-virus combination. Each combination was tested in duplicate, and IC50 values were averaged between the wells. Any IC50 values that were not reached at the lowest plasma dilution tested (1:20) were assigned an IC50 value of 10. Neutralization breadth scores were determined by adding the number of pseudoviruses in the panel neutralized at an IC50 greater than 20, while potency scores were determined by dividing the plasma-virus IC50 by the median IC50 per virus (against all plasma samples) and adding the scores for each plasma sample [10,48]. All potency score values were rounded to the nearest integer. In one case (ZM249M), because plasma was tested against an autologous envelope clone in the panel, IC50 values from this plasma-Env combination were discarded from the calculations for breadth and potency scores.

### gp120 binding ELISA

gp120 binding ELISAs were performed in triplicate as previously described [49]. Briefly, 96-well ELISA plates were coated overnight with 100 μl (2 μg/ml) purified gp120 protein (GeneART) from the Zambian subtype C seroconverter ZM205F [23,29] at 4°C. Plates were then washed six times with PBS-T (PBS containing 0.1% Tween-20) and blocked with 200 μl B3T (150 mM NaCl, 50 mM Tris–HCl, 1 mM EDTA, 3.3% FBS, 2% BSA, 0.07% Tween-20 in 500 ml ddH_2_0) for 1 hour at 37°C in a CO_2_-free incubator. Plates were washed again, and 100 μl/well of five-fold serially diluted heat-inactivated plasma was incubated for 1 hour at 37°C. After washing six times with PBS-T, 100 μl of diluted secondary antibody (HRP goat anti-human IgG) was added to each well for 1 hour at 37°C. After a final wash six times with PBS-T, 100 μl of SureBlue TMB substrate solution (equilibrated to room temperature) was added to each well. Plates were incubated for 10 minutes at room temperature. In order to stop the reaction, 100 μl of 1 N H_2_SO_4_ was added/well, and plates were read at 450 nm absorbance with a Biotek Synergy plate reader and luminometer. Wells coated with gp120 protein alone were used as blank control wells and were subtracted from absorbance readings, as described below in the Statistical analysis methods section.

This protocol was also adapted to measure plasma IgA levels, with the following changes: Test plasma was depleted of IgG using the GE Healthcare Protein G HP/Ab Spin Trap and was subsequently serially diluted five-fold at a starting concentration of 1:25 in B3T blocking buffer. Results shown at 1:125 plasma dilution are representative of the trends observed across the serial dilution. The secondary antibody was changed to an HRP-conjugated goat anti-human IgA antibody (InvivoGen). This assay was performed in duplicate with normal human plasma (NHP), autologous (ZM205F) 31-month plasma and Subtype C pooled plasma controls. Wells coated with gp120 protein alone were similarly used as blank control wells and were subtracted from absorbance readings.

### V1V2 binding ELISA

A standard ELISA protocol was used to evaluate the presence of V1V2-specific IgG binding antibodies in heat-inactivated patient plasma (diluted 1:500 in 2% BLOTTO). Plates were coated with MuLVgp70-caseA2clB V1V2 [30] or MuLVgp70-conC V1V2 (consensus clade C) scaffolded proteins with MuLVgp70 carrier alone as a control. Positive absorbance was defined as absorbance greater than five times that of the uninfected normal human plasma control.

### Statistical analysis

All statistical analyses compared responses between superinfected and non-superinfected groups. We performed the Wilcoxon rank sum test using the autologous neutralizing antibody IC50 titers obtained in the 5–8 month post-seroconversion time frame, which reflects the neutralizing antibody titers before superinfection. As subject ZM247F was superinfected at this time, for this subject alone we used the values obtained at 3 months post-seroconversion (which were equal to those obtained from 9- and 12-month plasma).

To evaluate differences in gp120-specific IgG binding antibody levels in pre-superinfection plasma in superinfected individuals and similar time points for controls, we evaluated gp120 binding ELISA data performed in triplicate. For each ELISA trial, we first found the baseline binding to purified gp120 protein – the lowest absorption value from the blank control wells. After adjusting for this baseline value, the experimental values were plotted, and the curve interpolated to find the titration corresponding to 50% of the highest binding absorption value of the curve. After all binding50 values were determined, we log-transformed the values for further analysis. A linear mixed effects model was used to determine whether the binding50 values were associated with superinfection status. Log-transformed binding50 values were used as the response variable, and the superinfection status was used as a predictor with fixed effect. The individual effects were modeled as random effects. We also calculated mean binding50 values for each test plasma and compared medians between superinfected and non-superinfected groups, using a Mann–Whitney test run in GraphPad Prism 5.0d. We similarly adjusted test plasma absorption values for background binding (as measured in the blank control wells) in the gp120-specific IgA ELISA, and compared median absorption values between groups amongst both trials using a Mann–Whitney test run in GraphPad Prism 5.0d.

## Competing interests

The authors declare that there are no competing interests.

## Authors’ contributions

The study was conceived and designed by: DB, CSK, CAD, EH. All experiments were performed by: DB, CSK, MKM, PJC. CI and AP generated the MuLVgp70-subtype B and consensus C V1V2 proteins. Data analysis was performed by EH, DB, CAD, TY, PTH. EC, JM, WK, SAA were critically involved in sample collection, site maintenance, participant recruitment and follow-up. All authors read and approved the manuscript. EH, CAD, MKM provided invaluable review and discussion of the project.

## Supplementary Material

Additional file 1 **Figure S1.** Radial neighbor-joining phylogenetic tree of full-length *env* amplicon sequences. This phylogenetic tree shows sequences of all full-length gp160 *env* amplicons isolated from time of seroconversion evaluated in this study in order to infer the subtype C early/founder Envs tested (red). Additional amplicons tested are shown in maroon, though no functional difference in neutralization phenotype between amplicons of the same patient was seen (data not shown). Sequences of Envs from the Subtype C HIV-1. Reference Panel of Env Clones panel [26,27] are also shown (blue). **Figure S2. Preferential neutralization of superinfecting virus Env is observed in one case of early intrasubtype C superinfection.** Autologous neutralizing antibody responses to both founder Env **(A)** and superinfecting Env **(B)** were measured over the first two years for ZM247F, in which superinfection was detected at 3-months post-seroconversion. Percent viral infectivity is depicted on the vertical axis and reciprocal plasma dilution is depicted along the horizontal axis, in logarithmic fashion. Each curve represents a single plasma-virus combination, performed in duplicate wells. Error bars represent standard error of the mean between two independent experiments. **Figure S3. Superinfecting viruses are sensitive to neutralization by pooled subtype C plasma.** We tested the ability of pooled subtype C plasma to neutralize superinfecting pseudoviruses from all three superinfected cases, in addition to SS1196.1 pseudovirus (carrying an envelope with Tier1b sensitivity) for comparison [27]. Percent viral infectivity is depicted on the vertical axis and reciprocal plasma dilution is depicted along the horizontal axis, in logarithmic fashion. Each curve represents a single plasma-virus combination, performed in duplicate wells. Error bars represent standard error of the mean between two independent experiments. **Figure S4. Limited heterologous neutralizing antibody breadth in superinfected individuals prior to superinfection.** Plasma from pre-superinfection (**A, B**) or early superinfection (SI), in the case of ZM247F **(C)**, time points was tested for heterologous neutralization to a subtype C Env reference panel. This panel included Envs of both Tier 1b and Tier 2 sensitivities [7,26]. Starting plasma dilution was reduced to 1:20 to increase assay sensitivity. Percent viral infectivity is depicted on the vertical axis and reciprocal plasma dilution is depicted along the horizontal axis, in logarithmic fashion. Each curve represents a single plasma-virus combination, performed in duplicate wells. Error bars represent standard error of the mean between two independent experiments. (PDF 545 kb) Click here for file
