# Supplementary material for: HIV-1 subtype C superinfected individuals mount low autologous neutralizing antibody responses prior to intrasubtype superinfection
Source: Retrovirology. 2012 Sep 20;9:76. doi: 10.1186/1742-4690-9-76 (PMC3477039; doi:10.1186/1742-4690-9-76)
Supplement: Additional file 1 — Figure S1. Radial neighbor-joining phylogenetic tree of full-length env amplicon sequences. This phylogenetic tree shows sequences of all full-length gp160 env amplicons isolated from time of seroconversion evaluated in this study in order to infer the subtype C early/founder Envs tested (red). Additional amplicons tested are shown in maroon, though no functional difference in neutralization phenotype between amplicons of the same patient was seen (data not shown). Sequences of Envs from the Subtype C HIV-1. Reference Panel of Env Clones panel [26,27] are also shown (blue). Figure S2. Preferential neutralization of superinfecting virus Env is observed in one case of early intrasubtype C superinfection. Autologous neutralizing antibody responses to both founder Env (A) and superinfecting Env (B) were measured over the first two years for ZM247F, in which superinfection was detected at 3-months post-seroconversion. Percent viral infectivity is depicted on the vertical axis and reciprocal plasma dilution is depicted along the horizontal axis, in logarithmic fashion. Each curve represents a single plasma-virus combination, performed in duplicate wells. Error bars represent standard error of the mean between two independent experiments. Figure S3. Superinfecting viruses are sensitive to neutralization by pooled subtype C plasma. We tested the ability of pooled subtype C plasma to neutralize superinfecting pseudoviruses from all three superinfected cases, in addition to SS1196.1 pseudovirus (carrying an envelope with Tier1b sensitivity) for comparison [27]. Percent viral infectivity is depicted on the vertical axis and reciprocal plasma dilution is depicted along the horizontal axis, in logarithmic fashion. Each curve represents a single plasma-virus combination, performed in duplicate wells. Error bars represent standard error of the mean between two independent experiments. Figure S4. Limited heterologous neutralizing antibody breadth in superinfected individuals prior [file 1742-4690-9-76-S1.pdf]

Supplementary Figure 1. Radial neighbor-joining phylogenetic tree of full-length *env* amplicon sequences.
This phylogenetic tree shows sequences of all full-length gp160 *env* amplicons isolated from time of seroconversion evaluated in this study in order to infer the subtype C early/founder Envs tested (red). Additional amplicons tested are shown in maroon, though no functional difference in neutralization phenotype between amplicons of the same patient was seen (data not shown). Sequences of Envs from the Subtype C HIV-1 Reference Panel of Env Clones panel [26, 27] are also shown (blue).

Supplementary Figure 2. Preferential neutralization of superinfecting virus Env is observed in one case of early intrasubtype C superinfection.
Autologous neutralizing antibody responses to both founder Env **(A)** and superinfecting Env **(B)** were measured over the first two years for ZM247F, in which superinfection was detected at 3-months post-seroconversion. Percent viral infectivity is depicted on the vertical axis and reciprocal plasma dilution is depicted along the horizontal axis, in logarithmic fashion. Each curve represents a single plasma-virus combination, performed in duplicate wells. Error bars represent standard error of the mean between two independent experiments.

Supplementary Figure 3. Superinfecting viruses are sensitive to neutralization by pooled subtype C plasma.
We tested the ability of pooled subtype C plasma to neutralize superinfecting pseudoviruses from all three superinfected cases, in addition to SS1196.1 pseudovirus (carrying an envelope with Tier1b sensitivity) for comparison [27]. Percent viral infectivity is depicted on the vertical axis and reciprocal plasma dilution is depicted along the horizontal axis, in logarithmic fashion. Each curve represents a single plasma-virus combination, performed in duplicate wells. Error bars represent standard error of the mean between two independent experiments.

Supplementary Figure 4. Limited heterologous neutralizing antibody breadth in superinfected individuals prior to superinfection.
Plasma from pre-superinfection (**A, B**) or early superinfection (SI), in the case of ZM247F **(C)**, time points was tested for heterologous neutralization to a subtype C Env reference panel. This panel included Envs of both Tier 1b and Tier 2 sensitivities [26, 27]. Starting plasma dilution was reduced to 1:20 to increase assay sensitivity. Percent viral infectivity is depicted on the vertical axis and reciprocal plasma dilution is depicted along the horizontal axis, in logarithmic fashion. Each curve represents a single plasma-virus combination, performed in duplicate wells. Error bars represent standard error of the mean between two independent experiments.

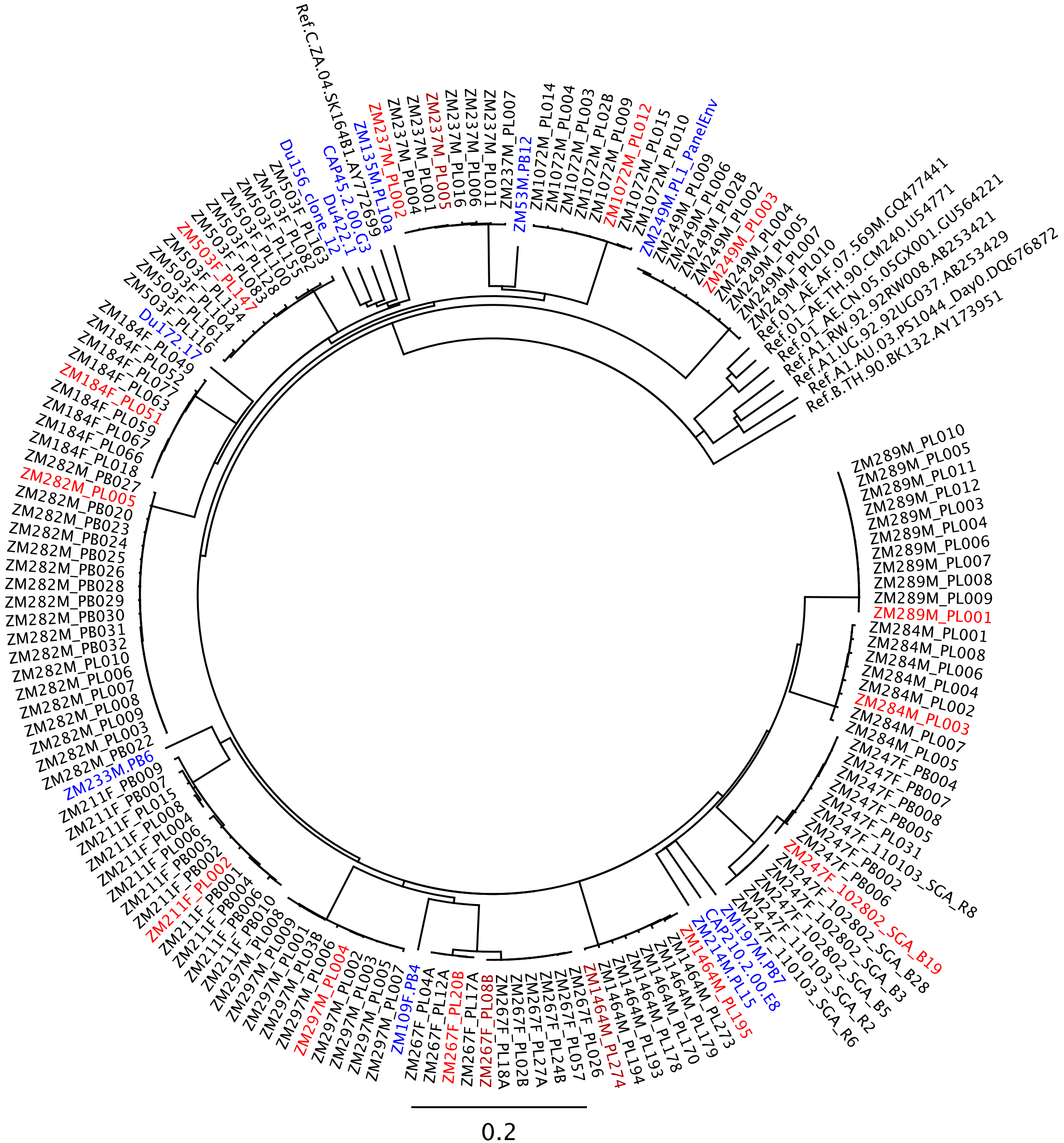

Ref.C.ZA.04.SK164B1.AY772699
ZM135M.PL10a
CAP45.2.00.G3
Du422.1
Du156_clone_12
ZM237M_PL002
ZM237M_PL004
ZM237M_PL001
ZM237M_PL005
ZM237M_PL016
ZM237M_PL006
ZM237M_PL011
ZM237M_PL007
ZM53M.PB12
ZM1072M_PL014
ZM1072M_PL004
ZM1072M_PL003
ZM1072M_PL02B
ZM1072M_PL009
ZM1072M_PL012
ZM1072M_PL015
ZM1072M_PL010
ZM249M.PL1_PanelEnv
ZM249M_PL009
ZM249M_PL006
ZM249M_PL02B
ZM249M_PL002
ZM249M_PL003
ZM249M_PL004
ZM249M_PL005
ZM249M_PL007
ZM249M_PL010
Ref.01_AE.AF.07.569M.GQ477441
Ref.01_AE.TH.90.CM240.U54771
Ref.01_AE.CN.05.05GX001.GU564221
Ref.A1.RW.92.92RW008.AB253421
Ref.A1.UG.92.92UG037.AB253429
Ref.A1.AU.03.PS1044_Day0.DQ676872
Ref.B.TH.90.BK132.AY173951
ZM289M_PL010
ZM289M_PL005
ZM289M_PL011
ZM289M_PL012
ZM289M_PL003
ZM289M_PL004
ZM289M_PL006
ZM289M_PL007
ZM289M_PL008
ZM289M_PL009
ZM289M_PL001
ZM284M_PL001
ZM284M_PL008
ZM284M_PL006
ZM284M_PL004
ZM284M_PL002
ZM284M_PL003
ZM284M_PL007
ZM284M_PL005
ZM247F_PB004
ZM247F_PB007
ZM247F_PB008
ZM247F_PB005
ZM247F_PL031
ZM247F_110103_SGA_R8
ZM247F_PB002
ZM247F_PB006
ZM247F_102802_SGA_B19
ZM247F_102802_SGA_B28
ZM247F_102802_SGA_B3
ZM247F_102802_SGA_B5
ZM247F_110103_SGA_R2
ZM247F_110103_SGA_R6
ZM197M.PB7
CAP210.2.00.E8
ZM214M.PL15
ZM1464M_PL195
ZM1464M_PL273
ZM1464M_PL179
ZM1464M_PL170
ZM1464M_PL178
ZM1464M_PL193
ZM1464M_PL194
ZM1464M_PL274
ZM267F_PL026
ZM267F_PL057
ZM267F_PL24B
ZM267F_PL27A
ZM267F_PL02B
ZM267F_PL18A
ZM267F_PL08B
ZM267F_PL17A
ZM267F_PL20B
ZM267F_PL12A
ZM267F_PL04A
ZM109F.PB4
ZM297M_PL007
ZM297M_PL005
ZM297M_PL003
ZM297M_PL002
ZM297M_PL004
ZM297M_PL006
ZM297M_PL03B
ZM297M_PL001
ZM297M_PL009
ZM297M_PL008
ZM211F_PB010
ZM211F_PB006
ZM211F_PB004
ZM211F_PB001
ZM211F_PL002
ZM211F_PB002
ZM211F_PB005
ZM211F_PL006
ZM211F_PL004
ZM211F_PL008
ZM211F_PL015
ZM211F_PB007
ZM211F_PB009
ZM233M.PB6
ZM282M_PB022
ZM282M_PL003
ZM282M_PL009
ZM282M_PL008
ZM282M_PL007
ZM282M_PL006
ZM282M_PL010
ZM282M_PB032
ZM282M_PB031
ZM282M_PB030
ZM282M_PB029
ZM282M_PB028
ZM282M_PB026
ZM282M_PB025
ZM282M_PB024
ZM282M_PB023
ZM282M_PB020
ZM282M_PL005
ZM282M_PB027
ZM184F_PL018
ZM184F_PL066
ZM184F_PL067
ZM184F_PL059
ZM184F_PL051
ZM184F_PL063
ZM184F_PL077
ZM184F_PL052
ZM184F_PL049
Du172.17
ZM503F_PL116
ZM503F_PL161
ZM503F_PL104
ZM503F_PL134
ZM503F_PL147
ZM503F_PL083
ZM503F_PL128
ZM503F_PL100
ZM503F_PL155
ZM503F_PL082
ZM503F_PL163
0.2

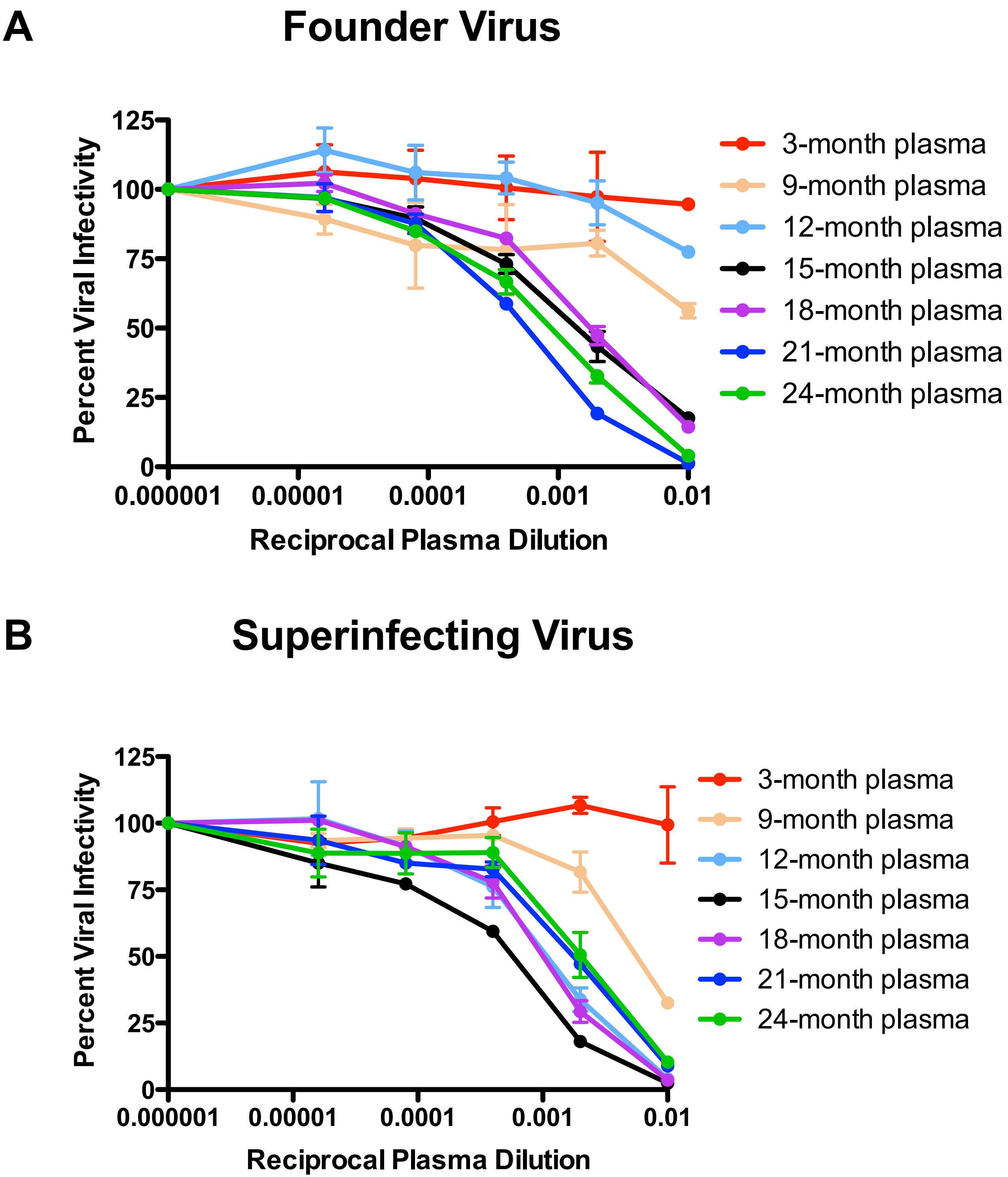
A
Founder Virus
Percent Viral Infectivity
125
100
75
50
25
0
0.000001
0.00001
0.0001
0.001
0.01
Reciprocal Plasma Dilution
3-month plasma
9-month plasma
12-month plasma
15-month plasma
18-month plasma
21-month plasma
24-month plasma
B
Superinfecting Virus
Percent Viral Infectivity
125
100
75
50
25
0
0.000001
0.00001
0.0001
0.001
0.01
Reciprocal Plasma Dilution
3-month plasma
9-month plasma
12-month plasma
15-month plasma
18-month plasma
21-month plasma
24-month plasma

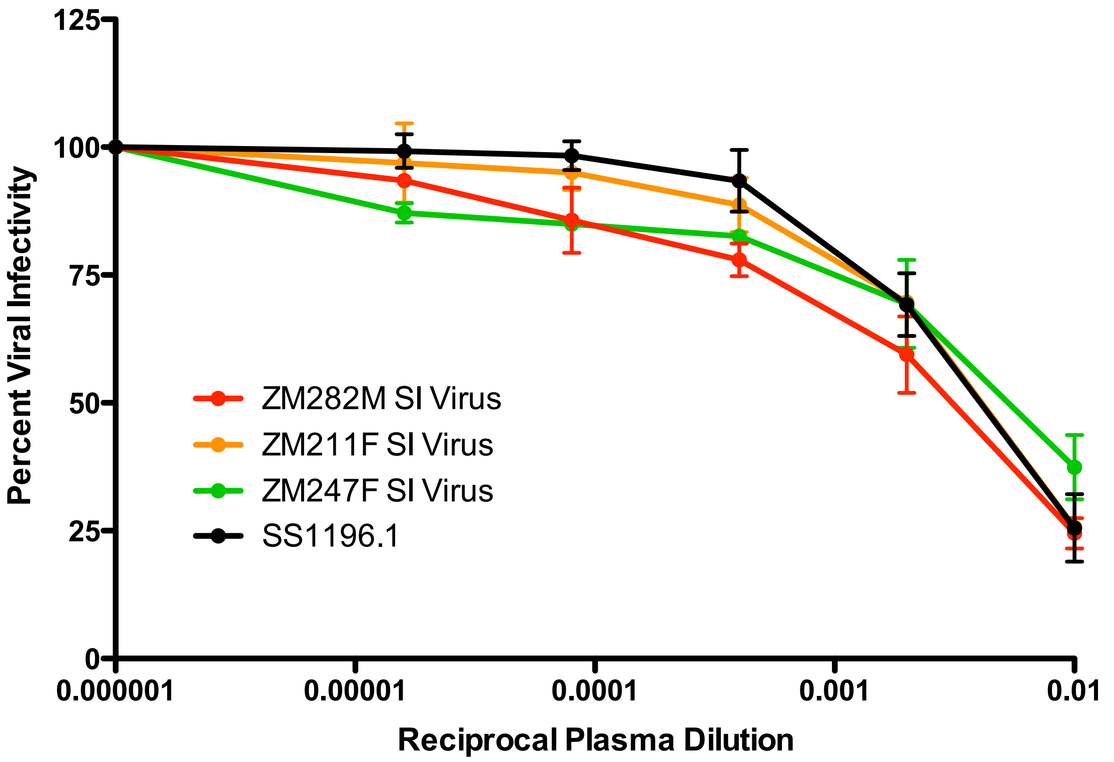

125
100
75
50
25
0
Percent Viral Infectivity
0.000001
0.00001
0.0001
0.001
0.01
Reciprocal Plasma Dilution
ZM282M SI Virus
ZM211F SI Virus
ZM247F SI Virus
SS1196.1

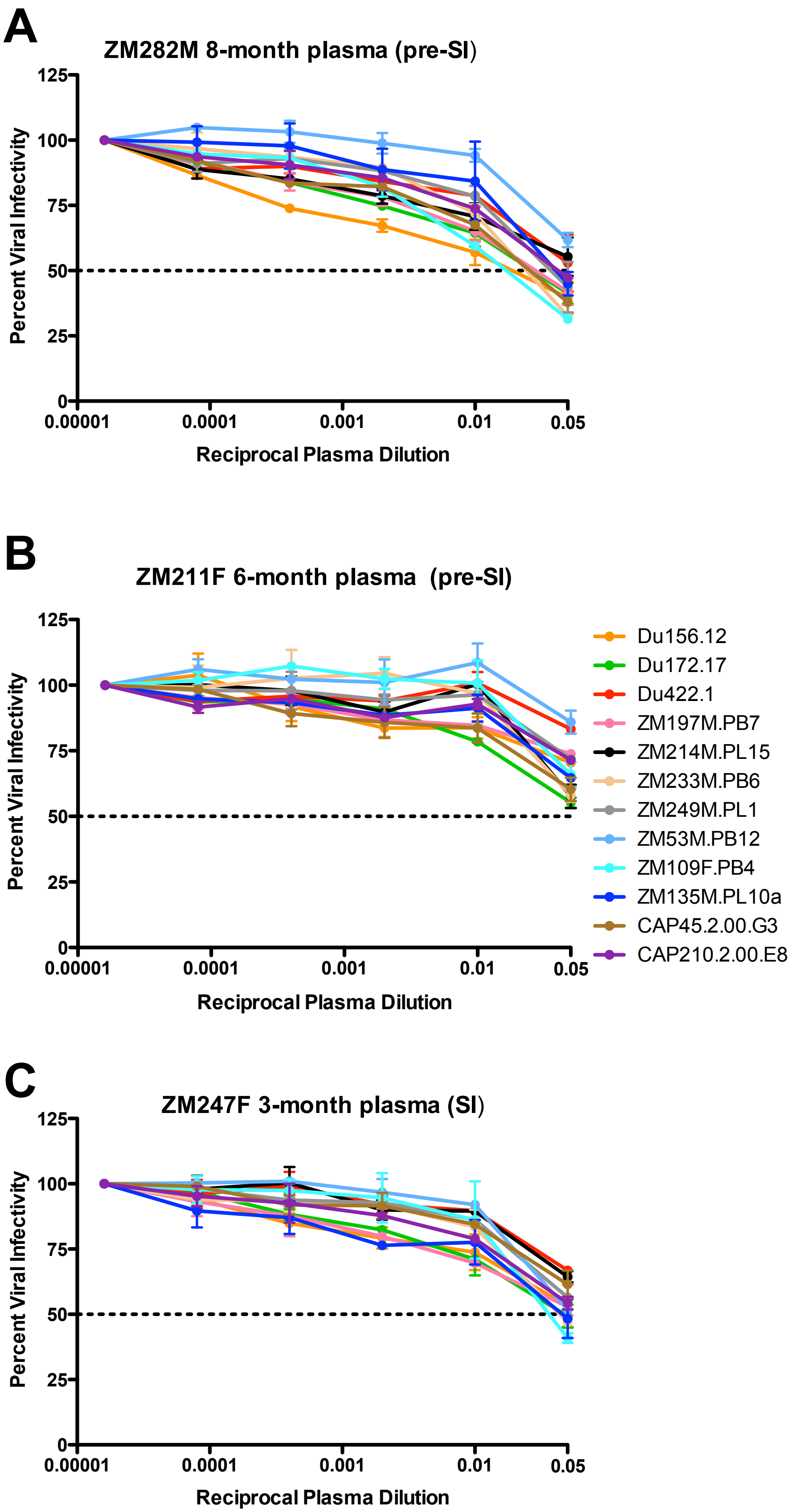

A
ZM282M 8-month plasma (pre-SI)
Percent Viral Infectivity
125
100
75
50
25
0
0.00001
0.0001
0.001
0.01
0.05
Reciprocal Plasma Dilution
B
ZM211F 6-month plasma (pre-SI)
Percent Viral Infectivity
125
100
75
50
25
0
0.00001
0.0001
0.001
0.01
0.05
Reciprocal Plasma Dilution
Du156.12
Du172.17
Du422.1
ZM197M.PB7
ZM214M.PL15
ZM233M.PB6
ZM249M.PL1
ZM53M.PB12
ZM109F.PB4
ZM135M.PL10a
CAP45.2.00.G3
CAP210.2.00.E8
C
ZM247F 3-month plasma (SI)
Percent Viral Infectivity
125
100
75
50
25
0
0.00001
0.0001
0.001
0.01
0.05
Reciprocal Plasma Dilution
